# Supplementary material for: Multi‐dataset identification of innovative feature genes and molecular mechanisms in keratoconus
Source: J Cell Mol Med. 2024 Sep 19;28(18):e70079. doi: 10.1111/jcmm.70079 (PMC11412914; doi:10.1111/jcmm.70079)
Supplement: Supplementary file 2 — Material S2. [file JCMM-28-e70079-s002.zip › 7.GSEA analysis/2.ATOH7/KEGG_STEROID_BIOSYNTHESIS.html]

Details for gene set KEGG\_STEROID\_BIOSYNTHESIS[GSEA]

|  || Dataset | ATOH7.group.cls#h\_versus\_l |
| Phenotype | group.cls#h\_versus\_l |
| Upregulated in class | l |
| GeneSet | KEGG\_STEROID\_BIOSYNTHESIS |
| Enrichment Score (ES) | -0.3562342 |
| Normalized Enrichment Score (NES) | -1.1134903 |
| Nominal p-value | 0.33333334 |
| FDR q-value | 1.0 |
| FWER p-Value | 0.995 |
Table: GSEA Results Summary

  

Fig 1: Enrichment plot: KEGG\_STEROID\_BIOSYNTHESIS      
 Profile of the Running ES Score & Positions of GeneSet Members on the Rank Ordered List

  

| SYMBOL | TITLE | RANK IN GENE LIST | RANK METRIC SCORE | RUNNING ES | CORE ENRICHMENT || 1 | SOAT1 | na | 3129 | 0.274 | 0.0513 | No |
| 2 | CYP27B1 | na | 3610 | 0.251 | 0.1670 | No |
| 3 | LIPA | na | 14699 | 0.024 | -0.1402 | No |
| 4 | FDFT1 | na | 18138 | -0.005 | -0.2366 | No |
| 5 | HSD17B7 | na | 18173 | -0.006 | -0.2347 | No |
| 6 | NSDHL | na | 20808 | -0.030 | -0.2953 | No |
| 7 | MSMO1 | na | 22304 | -0.046 | -0.3144 | No |
| 8 | DHCR24 | na | 23757 | -0.066 | -0.3221 | Yes |
| 9 | EBP | na | 24146 | -0.072 | -0.2963 | Yes |
| 10 | SQLE | na | 24502 | -0.077 | -0.2666 | Yes |
| 11 | CYP51A1 | na | 24506 | -0.077 | -0.2269 | Yes |
| 12 | TM7SF2 | na | 24585 | -0.079 | -0.1886 | Yes |
| 13 | SC5D | na | 25036 | -0.086 | -0.1571 | Yes |
| 14 | DHCR7 | na | 26300 | -0.111 | -0.1364 | Yes |
| 15 | LSS | na | 28333 | -0.160 | -0.1122 | Yes |
| 16 | SOAT2 | na | 31460 | -0.266 | -0.0648 | Yes |
| 17 | CEL | na | 32231 | -0.308 | 0.0719 | Yes |
Table: GSEA details [plain text format]

  

Fig 2: KEGG\_STEROID\_BIOSYNTHESIS      
 Blue-Pink O' Gram in the Space of the Analyzed GeneSet

  

Fig 3: KEGG\_STEROID\_BIOSYNTHESIS: Random ES distribution      
 Gene set null distribution of ES for **KEGG\_STEROID\_BIOSYNTHESIS**
